# Supplementary material for: Evaluation of Carbon Ion Radiation-Induced Trismus in Head and Neck Tumors Using Dose-Volume Histograms
Source: Cancers (Basel). 2020 Oct 25;12(11):3116. doi: 10.3390/cancers12113116 (PMC7693287; doi:10.3390/cancers12113116)
Supplement: Supplementary file 1 [file cancers-12-03116-s001.pdf]

Supplementary Materials: Evaluation of Carbon Ion Radiation-Induced Trismus in Head and Neck Tumors Using Dose-Volume Histograms

Atsushi Musha [1,2,*], Hirofumi Shimada [1], Nobuteru Kubo [1], Hidemasa Kawamura [1], Naoko Okano [1], Yuhei Miyasaka [1], Hiro Sato [1], Katsuyuki Shirai [3], Jun-ichi Saitoh [4], Satoshi Yokoo [2], Kazuaki Chikamatsu [5] and Tatsuya Ohno [1]

Table S1. Univariate analysis of risk factors for carbon ion radiotherapy-induced trismus.

Parameter	*n*	*p*-Value	Odds Ratio (95% Cl)
Age(years)			
59≦	14	0.517	1.846(0.285-11.978)
59＞	17		
Gender			
man	14	0.517	1.846(0.285-11.978)
woman	17		
Primary site			
Maxillary sinus and Nasal cavity	16	0.93	1.083(0.182-6.439)
others	15		
Histological type			
Adenoid cystic carcinoma	17	0.118	0.185(0.19-1.816)
others	14		
T stage			
T2-3	12	0.527	0.563(0.93-3.391)
T4	19		
GTV(Gross tumor volumes, cm3)			
28.68≦	16	0.411	2.167(0.334-14.057)
28.68＞	15		
